# Impact of the angle used in 2D Ultrasonography on the foetal femur diaphysis measurement.

**Published:** 2017-06

**Authors:** Anouck Lincé, Xavier Capelle, SyLvie Lepage, Frédéric Kridelka, Christine Van Linthout

**Affiliations:** Department of Gynaecology and Obstetrics, University of Liège, 4000 Liège, Belgium

**Keywords:** 2D ultrasound, Femur diaphysis, Foetal biometry, Quality score

## Abstract

**Objective:**

The purpose of this pilot study is to compare the 2D scanning measurement of the foetal femoral diaphysis using anterior or lateral/external incidence at ultrasound.

**Methods:**

In August 2016, 30 consecutive patients underwent a second trimester morphology ultrasound between 21 and 24 weeks of gestation by a senior sonographist. In each case, the femur length was measured either with an anterior angle, estimating the straight aspect of the diaphysis or with a lateral angle, assessing its curved aspect. The two measures were collected prospectively. The difference between paired measurements was calculated and expressed in percentage (mm) and in percentile.

**Results:**

The median difference between the two ultrasound angles in terms of femur length was 3,55% and in terms of percentile variation was 17,16.

**Conclusion:**

An anterior angle of measurement of the femur length seems to allow an optimal measure of the straight and longest aspect of the diaphysis. According to our results, this angle should be considered when scoring the quality of a morphological ultrasound, but further and larger studies should be done to confirm our hypothesis.

## Introduction

Sonographic foetal biometric measurements (biparietal diameter, head and abdominal circumference and femur length) are key variables used for an accurate determination of the gestational age, for detection of growth abnormalities and for estimation of the foetal weight. The foetal femoral length is also used for screening of Down syndrome and diagnosis of bones abnormalities.

Foetal biometry is determined upon standardized ultrasound planes and can be evaluated by an image-based scoring system (Salomon et al., 2006; Papageorghiou et al., 2013). The methodology with the least variations in measurement should be used (Chan et al., 2009).

The purpose of this study was to determine the potential variation of the femur length when using different (anterior vs lateral/external) angles of measurement.

## Materials and Methods

### Data collection

Femur diaphysis measures of 31 consecutive foetuses with no abnormalities noted at morphological ultrasound have been prospectively collected in August 2016. The data were obtained between 21 and 24 weeks of gestation by a senior and experienced sonographist.

We performed the measurements of the femur diaphysis using a two-step approach. First, the femur length was systematically measured with an anterior angle estimating the straight aspect of the diaphysis.

**Fig. 1:**
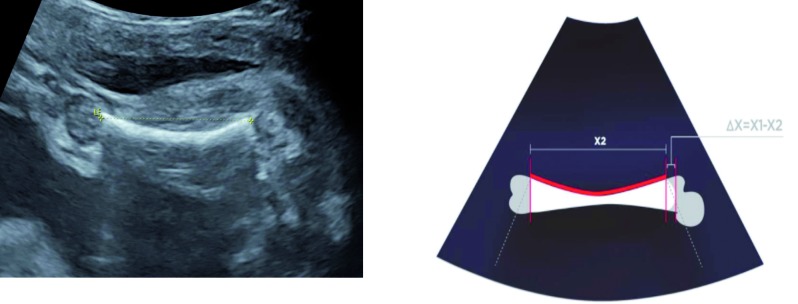
— Ultrasound scan of a femur diaphysis measured with an anterior angle. x1 = measure of the diaphysis length in mm.

**Fig. 2:**
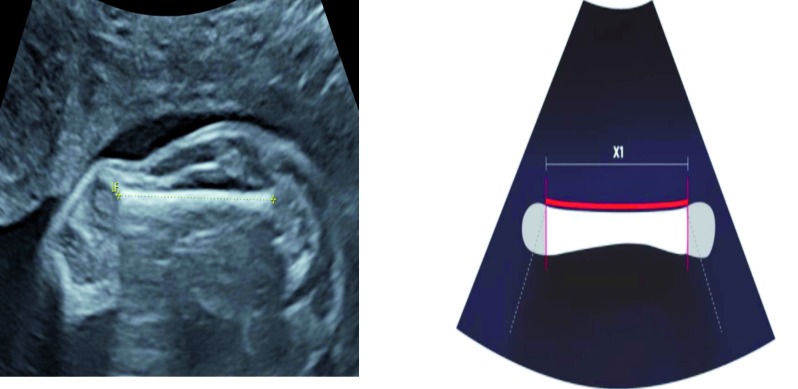
— Ultrasound scan of a femur diaphysis measured with a lateral/external angle. X2 = measure of the diaphysis length in mm. Delta x = x1 – x2.

Secondly, the femur diaphysis was measured with a lateral/external angle assessing its curved aspect (Fig. 1 and 2).

For each of the 62 measures, quality steps as defined in the literature have been respected; (1) a longitudinal view of the foetal thigh closest to the probe with the femur as close as possible to the horizontal plane (angle of < 45° to the horizontal), (2) both ends of the bone are clearly visible, (3) femoral plane occupying more than 30% of the total image size and (4) calipers placed correctly at both distal ends of the diaphysis ensuring that the trochanter is not included (Papageorghiou et al., 2013). The first metric measurements were blinded and the sonographer had only access to both measurements at completion of the examination.

All measures have been performed with a Voluson E10 Echograph using a curved electronic matrix abdominal 4D probe.

### Quality control

Two experienced foetal sonographers have reviewed each ultrasound scan and have assessed the quality of the measurement methods per the criteria published in the international literature. The two experts were blinded for the metric measurements. A single case didn’t fulfil all quality criteria and was excluded. A total of 30 femur measurements have been selected for the study.

### Anterior versus Lateral/External Measures

The differences between the measures obtained, respectively with the anterior and the lateral/ external ultrasound angle, are expressed in terms of percentage (difference of measures in mm divided by the median of measurements) and in terms of percentile.

## Results

**Fig. 3:**
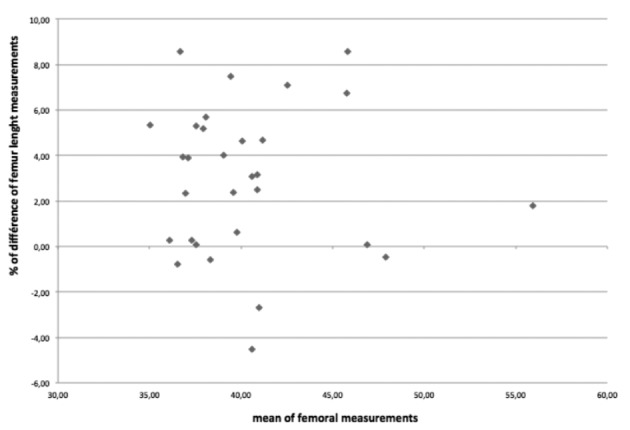
— Differences (%) of femur length compared to their mean

The mean difference in terms of femur length when assessed respectively by the anterior versus the lateral/external ultrasound angle was 3,55% (-4,51 to 8,56%) (Fig. 3). In 26 cases (84 %), the measure of the femur length is greater when assessed with the anterior angle. In 11/30 cases (36.6%) the difference is <1mm, in 13/30 cases the difference ranges between 1 and 2mm and the difference is >2mm in the remaining 6 cases (43%) for an average femur length at this gestational age of 40mm (P50 at 22 weeks).

**Fig. 4:**
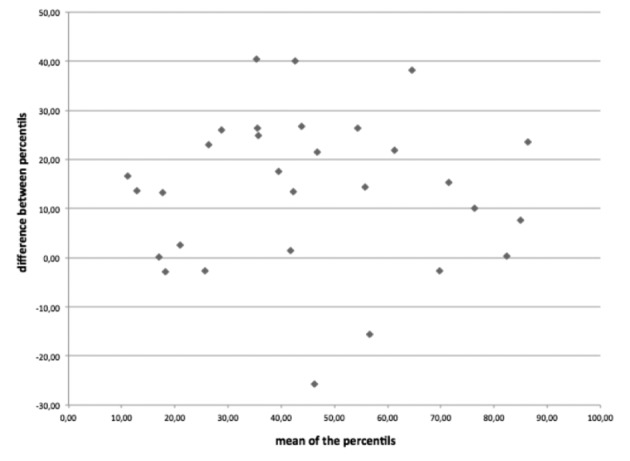
— Differences (Percentile) of femur length compared to their mean

In terms of percentile, a median difference of 17,16 is observed (Fig. 4). In two cases, the femur length is estimated < P 10 when the lateral/external angle (respectively P3 and P6) is used while the same femur length are within normal percentile range when evaluated with an anterior approach (respectively P19,5 and 19,6).

## Discussion

Accurate assessment of the femoral length is essential in order to diagnose bone and growth abnormalities.

In 2006, Salomon et al. (2006) proposed 4 criteria to be respected for a qualitative and reproducible femur measurement; the diaphysis must be addressed orthogonally with an angle < 45° to the horizontal, both epiphyses must be visible, the femur must occupy 30 to 50% of the image and the calipers must be positioned at both diaphysis extremities. A maximum score of 4/4 can be obtained and is associated with low intra- inter-observer variability (Salomon et al., 2006). However, the ultrasound angle with which the measurements are taken, is not mentioned and can clearly be of interest for a curvy bone such as the foetal femur. Despite this limitation, the quality criteria of Salomon are still used in the on-going prospective INTERGROWTH- 21st study (Papageorghiou et al., 2013; Stirnemann et al., 2017).

Indeed the femur length may vary according to the ultrasound angle used during an ultrasound evaluation. The superior and external extremity has a roundish aspect although the anterior extremity has a right angle with the bony femoral metaphysis. When the femur is measured with a lateral/external angle, the curved extremity is not fully exposed which participates to bias the estimated measure. At the opposite, when the femur is assessed using an anterior angle, only the straight aspect of the diaphysis is within the ultrasound range, while all other sides have a concave profile (Fig. 1 and 2).

Our study shows how the angle used in 2D ultrasound measurement of the femur length is critical with the lateral/external approach underestimating the bone length in 84% of the cases. This effect may have particular consequences amongst « short » femur which, if measured with a lateral/external approach may be classified as pathological (< P10) and wrongly suggest a growth deficit or a skeletal pathology.

Quality criteria for the measurement of the femoral length at prenatal ultrasound do not, to our knowledge, take the angle of analysis into consideration, may result in intra- and inter-observer variation. Moreover, in our study, the two angles of femur length analysis have been systematically obtained during a routine ultrasound examination. Within the context of our experience, the acquisition of both measurements did not encounter technical limitations, nor did it seem to prolong the duration of the ultrasound examination. Obtaining an optimal measurement of the femur length which respects the 4 published quality criteria as well as a reproducible and optimal angle of analysis, appears feasible and would strengthen the conclusion in terms of growth evaluation.

The small number of cases limits our study conclusions. The objective was to conduct a pilot study to verify if a difference of femur length, depending on the ultrasound angle used for its measure, could be suggested. A larger number of patients undergoing an ultrasound examination is needed to confirm our observation. We notice that, despite a quality score of 4, the femur length was estimated shorter with the « optimal » anterior angle when compared to the lateral/external one in 5 cases. This probably highlights the fact that the perfect angle of analysis was not obtained in all cases despite all efforts to respect a standardized and qualitative approach. Moreover, all measures have been obtained by a single senior sonographist and have been performed during the second trimester morphological examination. It will be interesting to make the same measures during the third trimester when the epiphysis ossification takes place.

In conclusion, we believe that a standardized method of measurement of the femur length at the time of the second trimester morphological ultrasound should be used and respect the quality criteria published by Salomon et al. (2006) as well as an anterior angle of analysis. We suggest a larger study to confirm or reject our results and to justify clinical changes in common practice if needed.
